# Bacterial Landscape of Bloodstream Infections in Neutropenic Patients via High Throughput Sequencing

**DOI:** 10.1371/journal.pone.0135756

**Published:** 2015-08-13

**Authors:** Peter Gyarmati, Christian Kjellander, Carl Aust, Mats Kalin, Lars Öhrmalm, Christian G. Giske

**Affiliations:** 1 Karolinska Institutet, Department of Microbiology, Tumor and Cell Biology, Nobels väg 16, Stockholm, Sweden; 2 Karolinska University Hospital, Department of Clinical Microbiology L2:02, Stockholm, Sweden; 3 Karolinska Institutet, Department of Medicine, Division of Hematology, Stockholm, Sweden; 4 Karolinska Institutet, Department of Medicine, Solna, Infectious Diseases Unit, Center for Molecular Medicine, Karolinska University Hospital, Stockholm, Sweden; 5 Karolinska Institutet, Department of Infectious Diseases, Stockholm, Sweden; Graz University of Technology (TU Graz), AUSTRIA

## Abstract

**Background:**

Bloodstream infection (BSI) is a common and potentially life-threatening complication in patients with hematological malignancies and therapy-induced neutropenia. Administration of broad spectrum antibiotics has substantially decreased the mortality rate in febrile neutropenia, but bacterial infection is documented in only one-third or fewer of the cases. BSI is typically diagnosed by blood culture; however, this method can detect only culturable pathogens.

**Methods:**

In the present study, a total of 130 blood samples from hematological patients receiving dose-intensive antitumoural treatment were subjected to 16S rRNA PCR and 62 of them were cultured. PCR positive samples were processed to high throughput sequencing by amplifying the V1-V3 regions of the 16S rRNA gene to obtain a full spectrum of bacteria present in BSI.

**Results:**

Five phyla and 30 genera were identified with sequencing compared to 2 phyla and 4 genera with culture. The largest proportion of bacteria detected by sequencing belonged to Proteobacteria (55.2%), Firmicutes (33.4%) and Actinobacteria (8.6%), while Fusobacteria (0.4%) and Bacteroidetes (0.1%) were also detected. Ninety-eight percent of the bacteria identified by sequencing were opportunistic human pathogens and 65% belonged to the normal human microbiota.

**Conclusions:**

The present study indicates that BSIs in neutropenic hosts contain a much broader diversity of bacteria, likely with host origin, than previously realized. The elevated ratio of Proteobacteria in BSI corroborates the results found in other systemic inflammatory diseases, such as inflammatory bowel disease or mucosal infections. This knowledge may become of value for tailoring antimicrobial drug administration.

## Introduction

Infection during neutropenia is one of the most common causes of mortality in patients receiving chemotherapy. Mortality rates vary between 5–11% depending on the co-existing conditions and can rise even higher if bacteremia is present [1]. The standard microbiological diagnostic method in febrile episodes in neutropenic patients is blood culture. Its positivity rate is dependent on whether the patient has received antibiotic prophylaxis or not, but typically bacteremia may be identified in around 7–17% of the patients on antibiotics and in 14–31% of those who are not on antibiotic treatment. Around 50–70% of the identified bacteria are Gram-positive organisms [1–3], the high number is probably at least partly related to the use of prophylactic antibiotics in many clinical settings. Febrile neutropenia is treated with empirically chosen broad-spectrum antibiotics and a more comprehensive identification of the incriminated microorganisms would have the potential to reduce antibiotic overuse by targeting only specific bacteria, a strategy which could reduce the generation of resistant strains.

A substantial proportion of bacteria cannot be cultivated [4–7]. Diagnostic methods used to diagnose bloodstream infections (BSI) are mostly limited to blood culture, which can detect only culturable pathogens, or to real-time PCR, which detects microorganisms pre-defined by primers [8]. By blood culture only a restricted range of pathogens may be identified, it might take several days before a positive result is indicated and large volumes of blood are needed to obtain optimal sensitivity, typically 20–40 ml/fever episode. Molecular methods, using 16S rRNA amplicon sequencing, have the potential to reveal pathogens present in BSIs, which may be undetected by culture-dependent methods. It requires ≤ 1 ml of blood and because it uses the variable regions of the 16S rRNA gene, identification of bacteria to genus or species level is possible [4, 6, 9].

High-throughput sequencing is a quickly growing field, and has helped to characterize microorganisms in several different habitats. Its expansion is powered by the development of high throughput sequencing techniques, allowing sequencing billions of reads in a few days’ time. Sequencing of the 16S rRNA gene is commonly used for culture-independent analysis, as this gene is universally present in bacteria, it is amplifiable by targeting conserved regions but also allows characterization of microbes through its variable regions. Although massively parallel sequencing makes species identification and estimating species abundance possible by its high coverage, targeting multiple regions of the 16S rRNA gene allows a more accurate identification of microorganisms [9]. In the present study, the variable V1-V3 regions were sequenced in blood samples from neutropenic patients with fever and suspected BSI.

This study aimed to characterize the bacterial content in blood samples of immunocompromised hematological patients in BSIs using high-throughput sequencing. Sequencing data were then compared with results from blood culture, the current gold standard for the diagnosis of BSIs.

## Materials and Methods

### Study population and sampling

Patients with hematological malignancies fit for dose intensive antitumoural treatment at the Hematology Center, Karolinska University Hospital in Stockholm, Sweden, were eligible for enrollment. Patients with acute myeloblastic leukaemia (AML) were included upon diagnosis whereas patients with other diagnoses could be asked to participate at any time points of the antitumoural treatment. Included patients were then sampled with two 4.5 mL EDTA tubes at different time point; 1) at diagnosis (only patients with AML), 2) at fever onset during neutropenia before intravenous broad spectrum antibiotic treatment was initiated, 3) follow-up samples to the fever-onset sample (only patients with AML), and 4) persisting fever during intravenous broad spectrum antibiotic treatment.

Samples were taken over a 1-year period (2013 March-2014 March). Data on white blood cell count (WBC), absolute neutrophil count (ANC), C-reactive protein (CRP) levels as well as age, gender and hematological diagnosis were extracted retrospectively from the patients' medical records. Samples were handled anonymously.

### Ethics statement

Written consents were obtained from all patients. All adult subjects provided written, informed consent, and a parent or guardian of any child participant provided written, informed consent on their behalf. The study (recordal 2012/1929-31/1) was approved by The Regional Ethical Review Board in Stockholm.

### Definitions

Fever was defined as a single oral temperature of ≥38.5°C or a temperature of >38.0°C persisting for >1 hour. Neutropenia was defined as a neutrophil count of ≤0.5 ×10^9^ cells/L, or a higher count with a predicted decrease to ≤0.5 ×10^9^ cells/L within 24 hours.

### Culture

Commercial BacT-Alert 3D system with 2–2 aerobic and anaerobic bottles was used (bioMérieux, Marcy l`Etoile, France). BSI was defined as an infection manifested by the presence of bacteria in at least one culture bottle, or at least two blood culture bottles with the same microorganism growing in the case of common skin contaminants.

### Sample preparation and sequencing

Blood samples for sequencing were drawn into sterile 4.5 ml Vacutainer (Becton Dickinson, Franklin Lakes, NJ USA) tubes, were kept at 4°C and processed to DNA extraction within 1–24 hrs. MolYsis Complete5 kit (Molzym Life Science, Bremen, Germany) was used to extract bacterial DNA following the manufacturer’s instructions with the following exceptions: 5 minutes were used for the final elution instead of 1, and samples were dissolved in 50 ul water instead of 100 ul. Positivity for the 16S rRNA gene was controlled by the 520F (AYTGGGYDTAAAGNG)-802R (TACNVGGGTATCTAATCC) primer pair [10] with 1x Phusion High Fidelity master mix (New England Biolabs, Ipswich, MA, USA) and 200 nM primer concentration. Reactions were incubated at 98C for 2 min, then 98C for 30 sec, 40C for 30 sec, 72 for 1min 30sec, cycled 35 times and incubated at 72C for 5 min. Amplicon sizes were controlled on a 2% agarose gel. No template controls (NTCs) were run with each set of samples and all DNA extraction reagents were tested for 16S rRNA PCR as well in order to investigate the possible contamination from the reagents used [11], but no detectable amplification was noted. Since longer 16S rRNA fragments result in more accurate identification [12], PCR positive samples were subjected to library preparation with the 27F (AGAGTTTGATCCTGGCTCAG)– 534R (ATTACCGCGGCTGCTGG) primer pair covering the V1-V3 regions of the 16S rRNA [10], and were processed to 2x300 bp paired end (PE) sequencing on an Illumina MiSeq instrument at GATC Biotech (Konstanz, Germany) as recommended by the manufacturer. In order to examine possible contamination originated from the human blood and/or the environment, a blood sample from a healthy donor and NTC samples were overamplified with 45 PCR cycles and were processed to Sanger sequencing. The resulting reads did not show significant (≥95%) similarity to any known bacteria when compared to the NCBI *nr/nt* database. Sequencing reads generated in this study were deposited to Sequencing Read Archives under experiment SRA:SRX668701, while background controls were deposited to NCBI GenBank under accession number KR152337-KR152338.

### Data analysis

Reads below Q20 and 246 bp, and PE reads that could not be merged (FLASH, [13]) were removed. Cd-hit [14] was used for clustering with 99% similarity. Chimeras were removed using UCHIME [15]. BLASTn was used for similarity search [16] with *e* ≤ 10^−6^ and minimum similarity set to 97%, with references from the Ribosomal Database project (RDP 11, [17]). Taxonomic classification was based on NCBI Taxonomy [18]. Numbers of reads within each cluster were used to calculate relative abundances. Identified genera and species were included in the study if they contained at least 0.5% of the total number of operational taxonomic unit (OTU)-assigned reads in each sample. The Qiime package [19–22] was used for phylogenetic analysis with FastTree 2.1.3 [23] using the Silva_111 reference database [24] and was visualized with FigTree v1.4.2. The *exclude_seqs_by_blast*.*py* was used to check human DNA contamination as part of the Qiime package.

## Results

### Clinical characteristics

A total of 33 patients were included in the study; 19 with AML and 14 with other highly malignant hematological diagnoses. In total 130 blood samples were collected; 27 from AML patients at diagnosis, 38 at fever onset, and 41 follow-up samples. A total of 24 samples were collected from patients with persisting fever during broad spectrum antibiotic treatment (S1 Table).

Ninety-two samples were from patients with AML as the underlying diagnosis (70.8%), acute lymphoblastic leukaemia for 21 samples (16.2%), acute promyelocytic leukaemia for 8 samples (6.2%), mantle cell lymphoma for 4 samples (3.1%), Burkitt lymphoma for 3 samples (2.3%), and diffuse large B-cell lymphoma for 2 samples (1.5%).

The average age of the total study population was 52.2 years ± 16.3 (mean ± SD, n = 130) with 40% females, WBC = 0.8 ± 2.1 (n = 87), ANC = 0.3 ± 0.9 (n = 59), CRP level = 75 ± 53 (n = 78).

In fever onset samples, the age of patients was 51 ± 17.9 (n = 38), 39.5% females, WBC = 1.2 ± 3.1 (n = 33), ANC = 0.4 ± 1.2 (n = 29), CRP = 51.6 ± 37.5 (n = 30).

In persisting fever samples, the age was 53.1 ± 15.5 (n = 24), 33.3% females, WBC = 0.3 ± 0.7 (n = 20), ANC = 0.1 ± 0.4 (n = 10), CRP = 117.8 ± 49.5 (n = 16).

In follow up samples, the average age was 50.3 ± 15.8 years (n = 41), 41.5% females, WBC = 0.8 ± 1.1 (n = 34), ANC = 0.2 ± 0.4 (n = 20), CRP = 75.6 ± 54.6 (n = 32).

### Positivity rates

A total of 130 blood samples were investigated with 16S rRNA PCR in this study and 65 of them with blood culture. Nineteen samples were positive by PCR out of 130 (14.6%) and 10 by blood culture out of 65 (15.4%), with 6 samples positive by both methods (S1 and S2 Tables). Positivity rate in fever onset samples (n = 38) was 23.7% (9/38) with PCR and 21.1% (8/38) with culture; in persisting fever samples (n = 24) the corresponding rates were 29.2% (7/24) and 8.3% (2/24); in follow up samples (n = 41) 7.3% (3/41) were positive with PCR and none with culture. In the 27 inclusion none was found to be positive with PCR. Accordingly, a total of 19 samples were positive by PCR and thereby processed to sequencing.

### Sequencing

**Fig 1:**
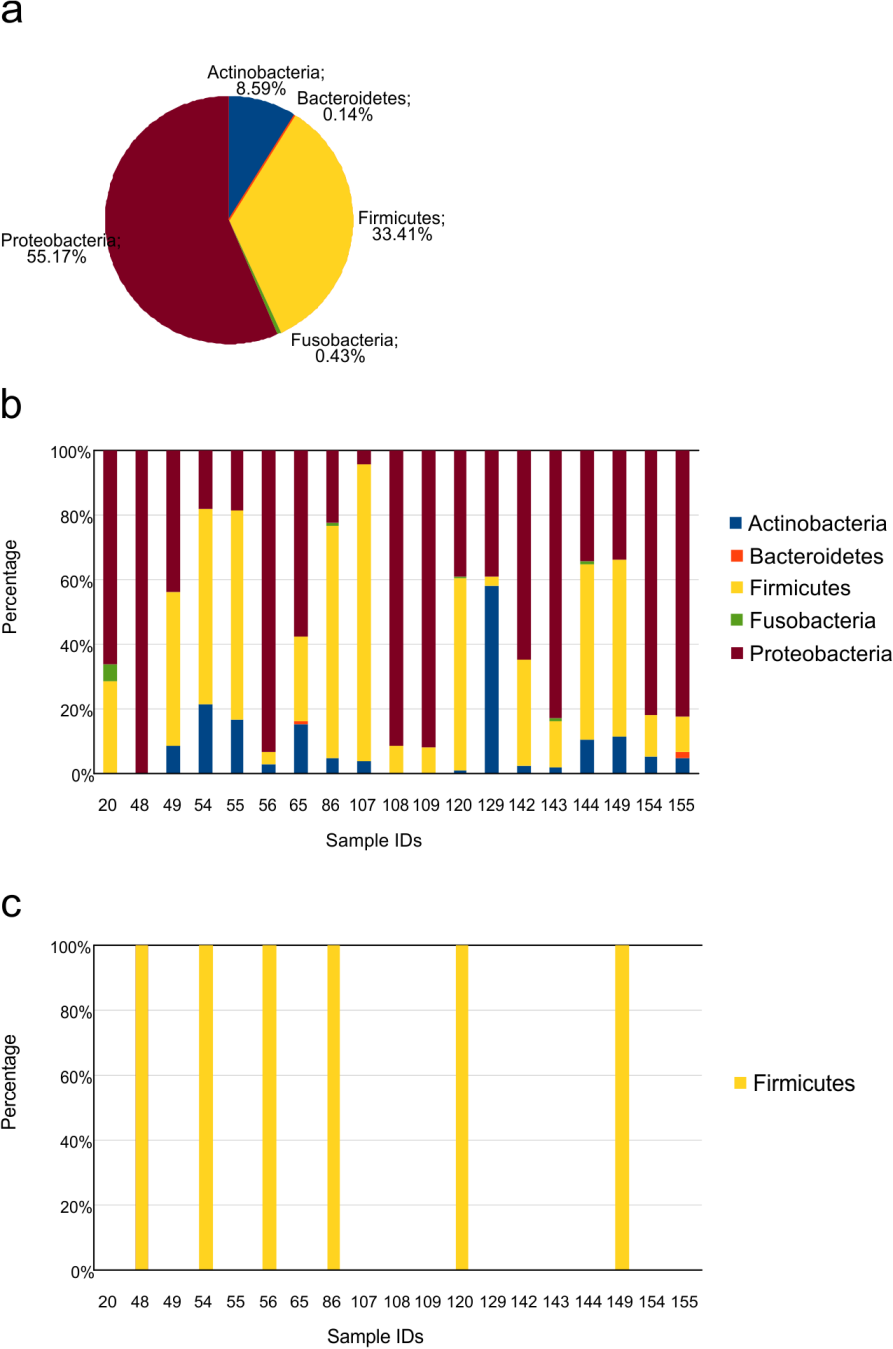
Representation of the distribution of phyla with sequencing in all samples (a) and in individual samples (b). Detected phyla per sample with blood culture (c).

PCR positive samples were processed to sequencing. A total of 2,764,592 reads were assigned to bacterial OTUs (S3 Table, average per sample: 145,504). In the entire dataset, sequencing detected members of five bacterial phyla; most reads were assigned to Proteobacteria (55.2%) and Firmicutes (33.4%). Apart from these, Actinobacteria (8.6%), Fusobacteria (0.4%) and Bacteroidetes (0.1%) were also detected (Fig 1). All samples except ID_48 contained bacteria from more than one phylum. Of the total number of reads, 55.7% belonged to Gram-negative bacteria.

**Fig 2:**
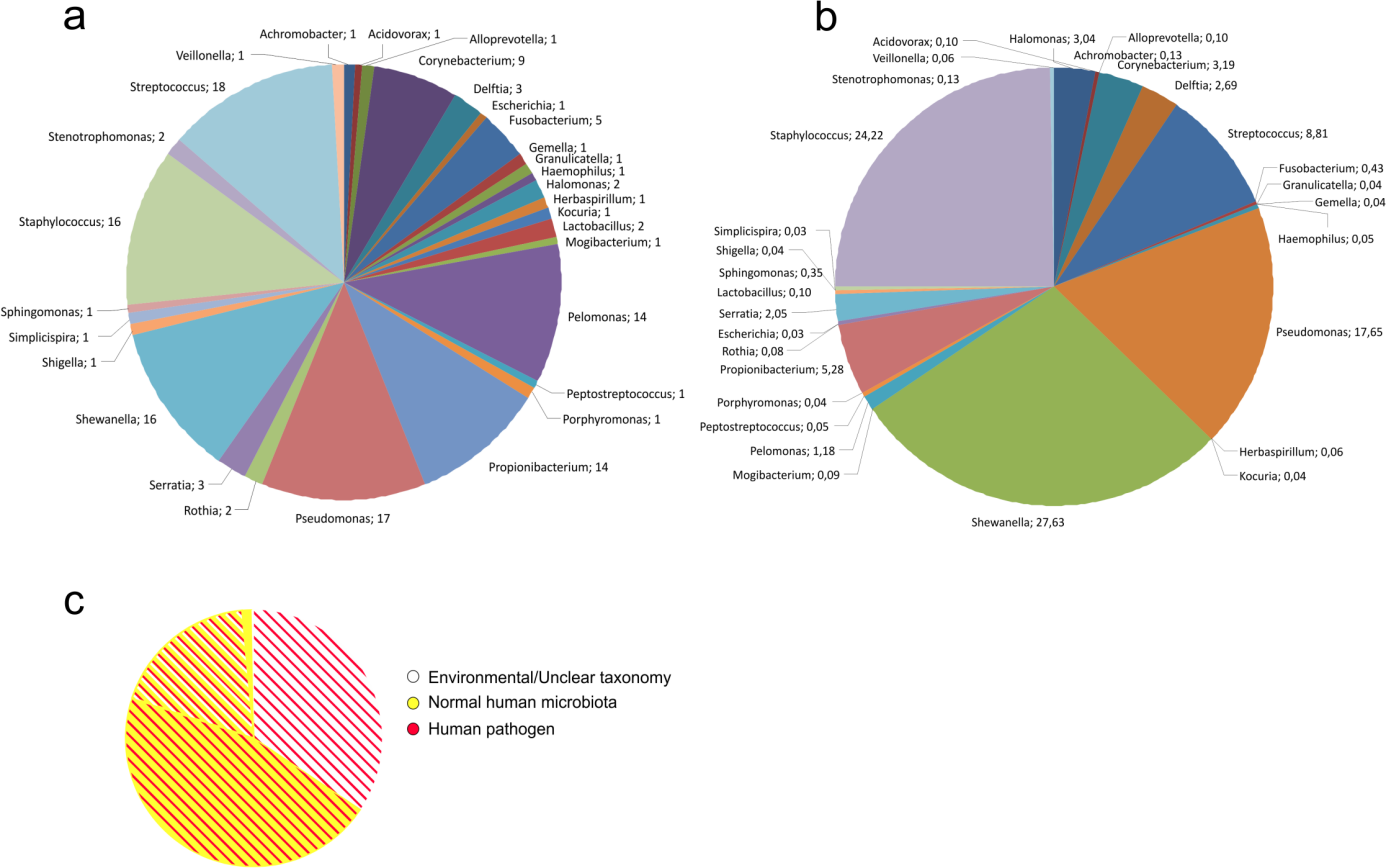
Occurrence of genera in 19 samples identified by sequencing (a). A genus was included if it reached or exceeded 0.5% of the total number of filtered reads in at least one sample. Distribution of OTU-assigned reads per genera in all samples in percentage (b). Diagram shows the pathogenicity and natural habitat of the detected genera based on read percentages (c). Over 96% of the identified reads belonged to opportunistic human pathogens (black stripes), while 64% belonged to the normal human microbiota (grey background).

Within the 5 phyla, 30 genera were identified, where *Streptococcus* (detected in 18 cases out of 19), *Pseudomonas* (17/19), *Shewanella* and *Staphylococcus* (16/19), *Pelomonas* and *Propionibacterium* (14/19) were the most prevalent. Sixteen genera occurred in only 1 case (Fig 2A) and 20 genera had <1% of all assigned reads (Fig 2B). Although *Streptococcus*, *Propionibacterium* and *Pelomonas* were amongst the most commonly occurring genera (detected in 18/14/14 cases, respectively; Fig 2A), the total read percentages show that they contain a relatively small proportion of all assigned reads (*Streptococcus*: 8.8%, *Propionibacterium*: 5.3%, *Pelomonas*: 1.2%; Fig 2B). On the other hand, *Delftia* and *Halomonas* genera occurred only in 3 and 2 cases, respectively, but contained 2.7% and 3% of all assigned reads.

**Fig 3:**
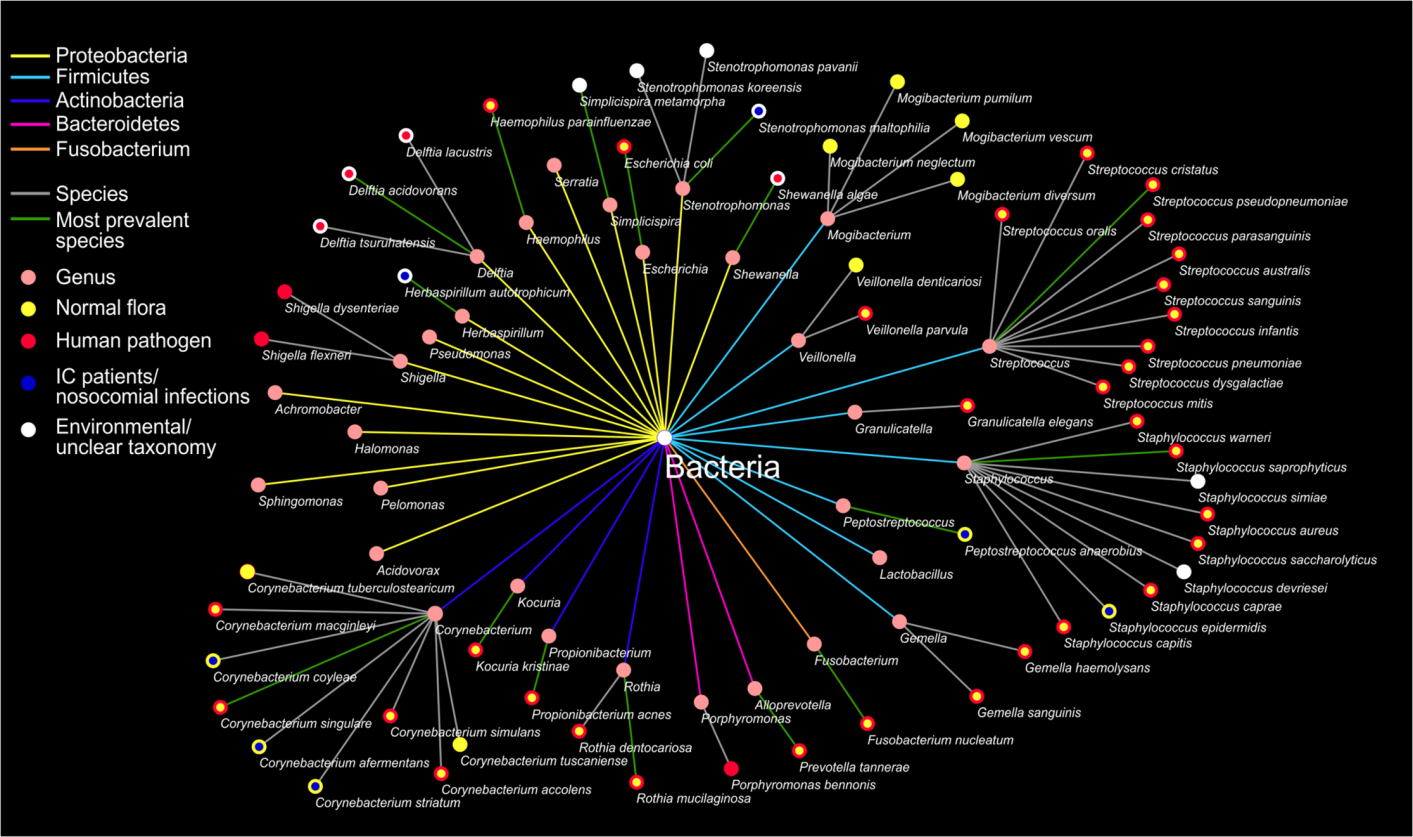
Schematic representation of microorganisms detected by sequencing on the species level. Pink nodes represent the given genera connected to the species. Green lines indicate the most prevalent species. Color lines from Bacteria to genera indicate phyla (Proteobacteria-yellow, Firmicutes-light blue, Actinobacteria-dark blue, Bacteroidetes-purple, Fusobacterium-orange). Species nodes indicate infectious properties: yellow-normal microbiota, red-human pathogen, blue-typically occurs in immunocompromised patients and/or nosocomial infections, white: taxonomy unclear/recently changed or environmental bacteria.

Over 98% of the identified reads belonged to reported human pathogens, and 65% of them belonged to the normal human microbiota (Fig 2C). Most of the identified genera (80.5% of the total reads) belonged to anaerobic or facultative anaerobic bacteria. Altogether 58 species were identified; 16 genera contained multiple species, typically with one dominant (Fig 3). Even though species were identified with ≥97% similarity, the 16S rRNA gene can have ≤1% diversity in between some species [9]. Eight genera could not be classified to the species level due to the high inter-species similarity. Sequencing of the V1-V3 regions also enabled phylogenetic analysis (S1 Fig).

The diversity of the samples illustrated with rarefaction curves (S2 Fig) indicate that in some cases >10,000x coverage might be necessary to identify all pathogens present with high-throughput sequencing.

### Culture

Viridans group streptococci (7), coagulase-negative staphylococci (CoNS, 2), *E*. *coli* (1) and *Enterococcus faecalis* (1) were identified in blood cultures, with one polymicrobial infection (CoNS + *E*. *faecalis*, in sample ID_86). All of the bacteria detected by both culture and sequencing belonged to the Firmicutes phylum (Fig 1C, S2 Table).

### Effect of antibiotic treatment on bacterial composition

**Fig 4:**
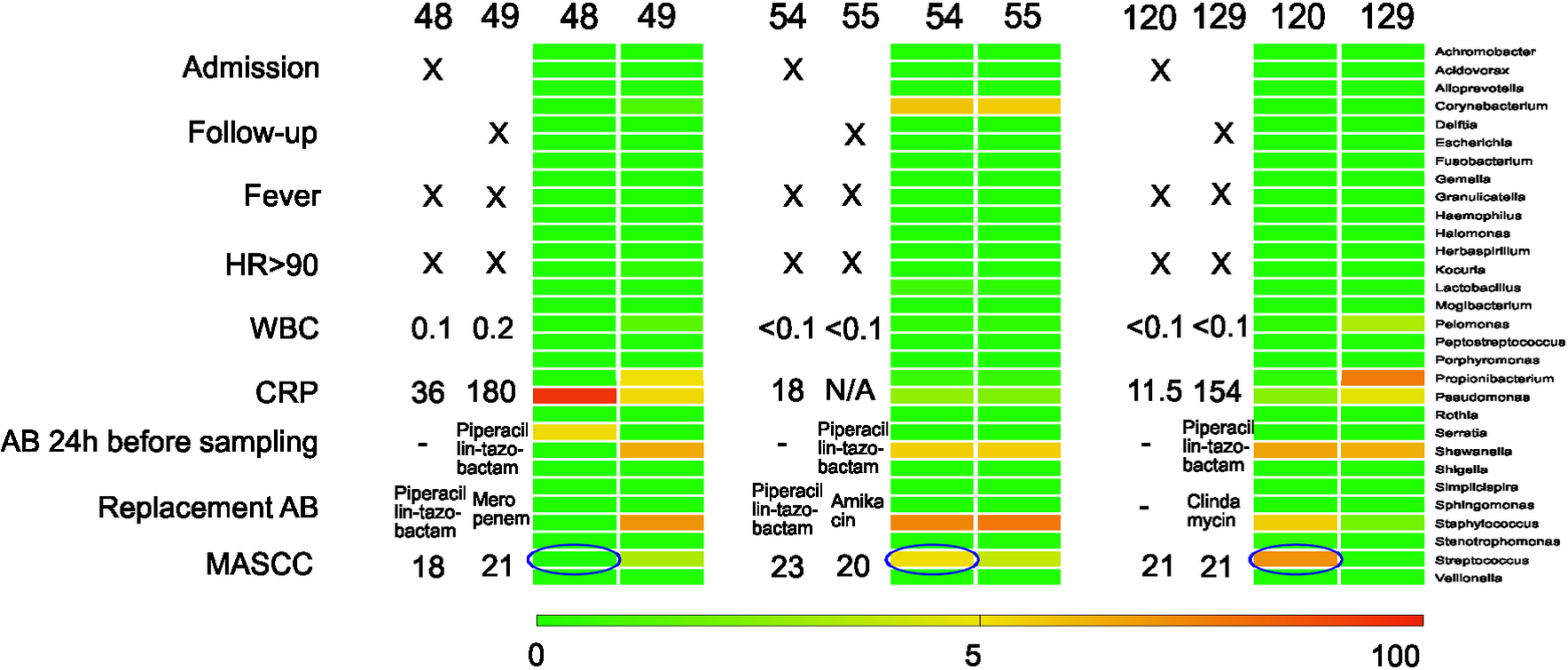
Heat map showing 3 pairs of samples before and after antibiotic treatment with the corresponding clinical characteristics (follow up: 1 day for samples 48/49 and 54/55, 5 days for samples 120/129). Bacteria were detected in all cases, but in 2 pairs (48–49, 120–129), the composition of bacteria changed, while in one patient (54–55), the composition of bacteria remained the same despite of antibiotic treatment. Blue ellipses indicate culture results. N/A: not available.

In three pairs of samples (before-after antibiotic treatment: samples 48–49, 54–55, 120–129) we found PCR positive samples despite of antibiotic treatment. In two cases (48–49 and 120–129) the bacterial composition underwent a major change after the antibiotic treatment, but in one case (54–55) the bacterial composition remained identical (Fig 4).

## Discussion

Febrile neutropenia is a severe medical condition in immunocompromised patients and in those undergoing chemotherapy; and is a common cause of death when coupled with bacteremia [25].

Routine diagnosis of BSIs is based on the identification of pathogens by use of blood culture bottles. However, blood cultures have several limitations: the growth in the bottle can be slow, and several days may be required before growth can be noted; a large volume of blood is required to optimize sensitivity and only culturable pathogens can be detected. In one study, high-throughput sequencing was proven to detect more bacterial pathogens and was shown to be more sensitive than culture or Sanger sequencing in CSF samples [26]. In the presented work, blood culture was shown to detect fewer microorganisms in fewer cases compared to high-throughput sequencing. Thus, results obtained with blood culture may not reveal optimal data for management and might lead to inadequate treatment. The Firmicutes phylum were dominant with blood culture (Fig 1C), indicating a narrow range of detectable pathogens, possibly due to a competition in the growth of culturable pathogens in blood cultures. Blood culture typically detects only one pathogen per sample, while the bacterial composition of BSIs in neutropenic febrile hosts seems to be much wider according to the results from high-throughput sequencing (Figs 1–3). It has to be noted that the efficacy of both classical and molecular diagnostics methods depends on several factors regarding the detection of pathogens in blood, including sampling, bacterial load, bacterial interference, etc.

Despite its numerous advantages, high-throughput sequencing raises considerable challenges as well: although sequencing costs continue to decrease, the cost of an instrument and the reagent costs remain high. Sequencing runs can take a few days to complete and the large amount of data generated from a sequencing run requires bioinformatics solutions. Due to sequence similarities of the 16S rRNA genes between microorganisms, identification of lower taxonomic categories (eg., species level) can be less certain [27] and antibiotic resistance patterns cannot be identified with this method. Also, while blood culture detects only viable microorganisms, pathogens identified by 16S rRNA sequencing might not necessarily be functional as shown in this study: positivity rate was the highest in fever onset samples both with PCR and culture (26% and 21%, respectively), indicating the presence of a high load of viable bacteria. However, in persisting fever samples, as the antibiotic treatment started, positivity rate of culture decreased to 4% while PCR maintained 24%, implying the presence of non-viable bacteria. PCR therefore offers an extended time for detecting BSIs during and after antibiotic treatment (S1 Table).

### High-throughput sequencing

Five phyla and thirty genera were identified with this method. All genera found in these samples have been previously reported in bacteremia except the *Pelomonas* genus, which, however, has been isolated from haemodialysis water [28]. The majority of sequencing reads belonged to bacteria which form the normal human microbiota (Fig 2C), supporting the notion that translocation of the human microbiota plays a decisive role in bacteremia [29]. In addition, mostly anaerobic bacteria were detected in the presented samples and similarly, the human microbiota largely consists of anaerobic bacteria [7].

The *Shewanella* genus (formerly classified as *Pseudomonas*) was detected in over 80% of the samples (Fig 3). Although *Shewanella* bacteremia is a well-reported phenomenon [30–33], our results suggest that its relevance may be underestimated as it is not routinely diagnosed. Studies suggest that especially immunocompromised patients might be commonly infected with this pathogen, although its clinical significance is not fully known [33]. Additionally, because of their different clinical characteristics and susceptibilities to antimicrobial agents, it is important to differentiate *S*. *algae* from *S*. *putrefaciens* and as shown, sequencing of the 16S rRNA gene can identify *Shewanella* on the species level.

Similarly to another report [34], *Staphylococcus* and *Pseudomonas* were amongst the most commonly identified pathogens in patients with neutropenia, although *Escherichia* was identified in only one case in the present report in contrast to the findings reported by Ortega *et al*. [34]. *Pseudomonas* commonly occurs in hospital-acquired infections in immunocompromised patients [35]; it is one of the most genetically divergent genera and it was one of the most prevalent genera in the examined samples. However, the similarity of the 16S rRNA gene between *Pseudomonas* strains can be >99% [36]; therefore sequencing of the 16S rRNA gene does not discriminate appropriately between species for the *Pseudomonas* genus [37, 38].

### The microbiota composition of BSI shows highest similarity to that of inflammatory bowel disease

**Fig 5:**
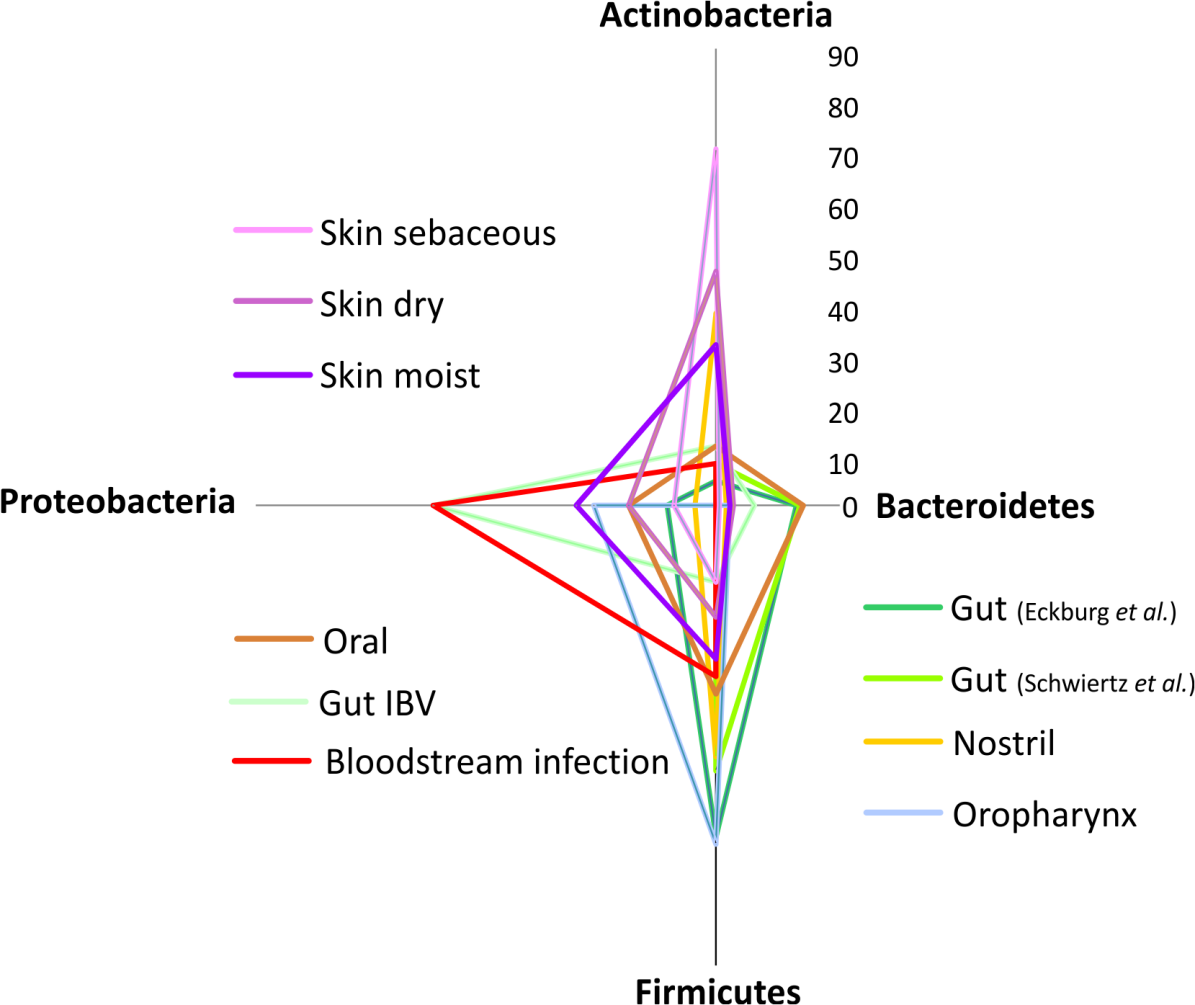
Composition of microbiota from different parts of the body classified by four major bacterial phyla. The graph was reconstructed based on the data from [44, 45, 51, 53, 55]. Axes show percentages.

The composition of microbiota can change in pathophysiological conditions associated with systemic inflammation, such as allergy or autoimmune diseases, due to the microbiota’s ability to participate in the regulation of the host’s immune system [39–43]. In order to investigate bacterial composition in BSIs, we compared the distribution of the four major phyla found in the reported samples with other studies characterizing the microbiota in different parts of the body (Fig 5).

In comparison to the microbiota in BSIs, Firmicutes and Bacteroidetes are the main phyla in the gut, while Proteobacteria can be found in very low percentages ([44, 45], Fig 5). However, gut microbiota from inflammatory bowel disease [44] showed the largest overlap with our samples from all compared microbiota, indicating that the formation and composition of microbes play an important role in systemic inflammation [39], represented by an increased proportion of Proteobacteria as demonstrated in the present study and in other cases [46–49].

On the skin, Firmicutes, Proteobacteria and Actinobacteria are also commonly found [50], although that depends on various factors, such as dryness of the skin and sampling sites [51]. Typically, the skin is dominated by Actinobacteria [52], and the moist skin sites had the largest overlap with our samples amongst different skin microbiota, possibly due to sampling or translocation [40]. Grice et al. reported a larger proportion of Proteobacteria when the skin was sampled from the inner elbow [52].

Lemon and colleagues reported [53] an inverse correlation between Actinobacteria and Firmicutes in the microbiota of the nostril. This effect has been detected in the presented samples as well, e.g., in samples ID_129 (with *Propionibacterium* detected) and 120 (with *Streptococcus* detected), where sample 120 represent the situation before antibiotic treatment.

Our results show partial similarity with the lung microbiota [40, 54], where, based on multiple studies, Proteobacteria and Firmicutes are consistently the most commonly identified phyla and *Pseudomonas*, *Streptococcus* and *Prevotella* are the most common genera. It has to be noted however, that methodologies for characterizing microbiota vary widely which might influence any comparison. Apart from the methodological aspects, microbiota carry-over might also be considered in clinical samples [40].

Bacteria identified in BSIs are dominated by Proteobacteria (Figs 1 and 5)–a phylum, which has been identified in local inflammations and has been recently associated with systemic inflammation [46–49]. The elevated ratio of Proteobacteria might be caused by the special metabolism of this phylum to utilize nitric metabolites abundant on inflammatory sites [39]. A large proportion of the identified bacteria belonged to the normal human microbiota (Figs 2 and 3), implying its role in the formation of systemic inflammatory response.

### High-throughput sequencing as a potential tool to assess the efficacy of antibiotic treatments

Characterization of the microbiota in BSIs would not only help in choosing antibiotic treatment options, but it would also enable to estimate the efficacy of antimicrobial treatment (Fig 4, S3 Fig). Interestingly, we could detect different effects of the antibiotic treatments in different samples of the same patient. In one case the content of bacteria did not change while in another case drastic changes could be observed. The former indicates that treatment did not eliminate the invading microorganisms, while in the latter case, elimination of the bacteria led to re-population or co-infection by different strains. One patient had *Pseudomonas* and *Serratia* detected before sampling (Fig 4, sample 48), and after piperacillin-tazobactam treatment, the proportion of these genera decreased, indicating the effect of antibiotic treatment consistent with these genera often being susceptible to this compound in our clinical setting. However instead *Propionibacterium* and *Staphylococcus* were present in the follow-up sample (sample 49), which could also be related to contamination from the skin microbiota. In samples 54 and 55, *Staphylococcus*, *Shewanella* and *Corynebacterium* were detected both before and after piperacillin-tazobactam treatment, possibly due to resistance to this drug. In samples 120 and 129, *Shewanella* prevailed, while *Staphylococcus* and *Streptococcus* disappeared after drug administration, which could be consistent with the administered combination treatment of piperacillin-tazobactam and clindamycin treatment.

Although these preliminary findings are based on a very limited number of samples, the data indicate that high-throughput sequencing may have the potential to become a promising tool in evaluating the efficacy of antibiotic therapy.

## Conclusion

Promoting rational antimicrobial use is essential to restrict the development of antibiotic resistance. As shown, high-throughput sequencing is able to identify a wide range of pathogens undetected by classical methods. By knowing the relative abundance of pathogens, a more customized treatment could be administered. Additionally, revealing the composition of microbiota in BSIs might help to understand its role in the pathomechanisms behind sepsis and provide information on the factors relevant in systemic inflammatory responses.

## Supporting Information

S1 Fig Phylogenetic representation of sample ID_149 showing distribution of the identified genera and phyla based on the assigned OTUs. (TIF)

S2 Fig Rarefaction curves show species count in relation to number of reads in OTU-assigned unique clusters. (TIF)

S3 Fig Heat map with a correlation scale shows the distribution of genera per sample. Blue ellipses indicate culture results.
(TIF)

S1 Table Representation of samples used in this study, cells indicating PCR / culture results, respectively. Brown = PCR positive, yellow = culture positive, green = positive with both methods, na = no culture taken, NA = no sample taken, sum = sum of positive samples. Empty cells = no sample available.
(DOCX)

S2 Table Comparison of bateria detected by sequencing and blood culture. ^1^ In patients 9 and 25, sequencing identified *S*. *mitis*, *S*. *pneumoniae* and *S*. *pseudopneumoniae*, while, apart from these three species, in patient 27 *S*. *infantis*, *S*. *oralis* and *S*. *australis* were also detected, confirming the presence of viridans streptococci. ^2^In patient 10, *S*. *dysgalactiae* (β-hemolytic streptococci) was detected by sequencing. ^3^In patient 17, in agreement with the culture result, sequencing detected a coagulase-negative Staphylococcus (*S*. *Saphrophyticus*). CoNS: Coagulase-negative staphylococci.
(DOCX)

S3 Table Sequencing statistics of assigned reads per sample. (DOCX)
