# Supplementary figures and images for: Bacterial Landscape of Bloodstream Infections in Neutropenic Patients via High Throughput Sequencing
Source: PLoS One. 2015 Aug 13;10(8):e0135756. doi: 10.1371/journal.pone.0135756 (PMC4536222; doi:10.1371/journal.pone.0135756)

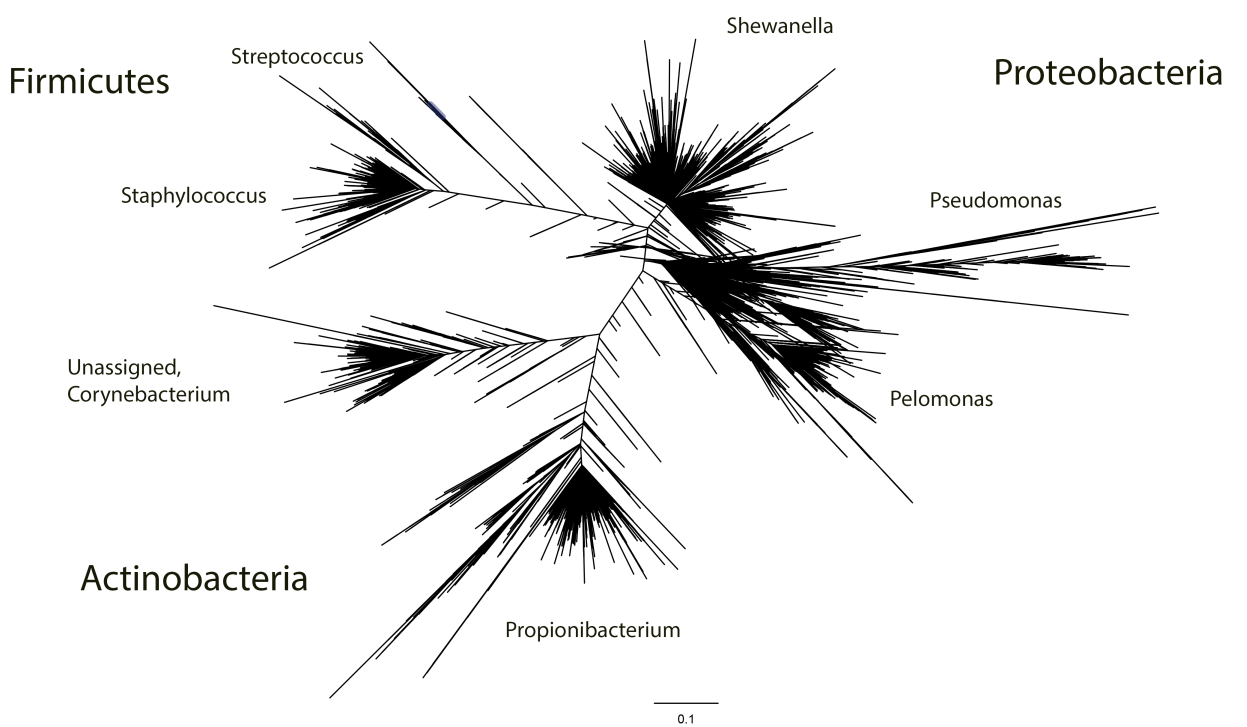

Supplement: S1 Fig — (TIF) [file pone.0135756.s001.tif]

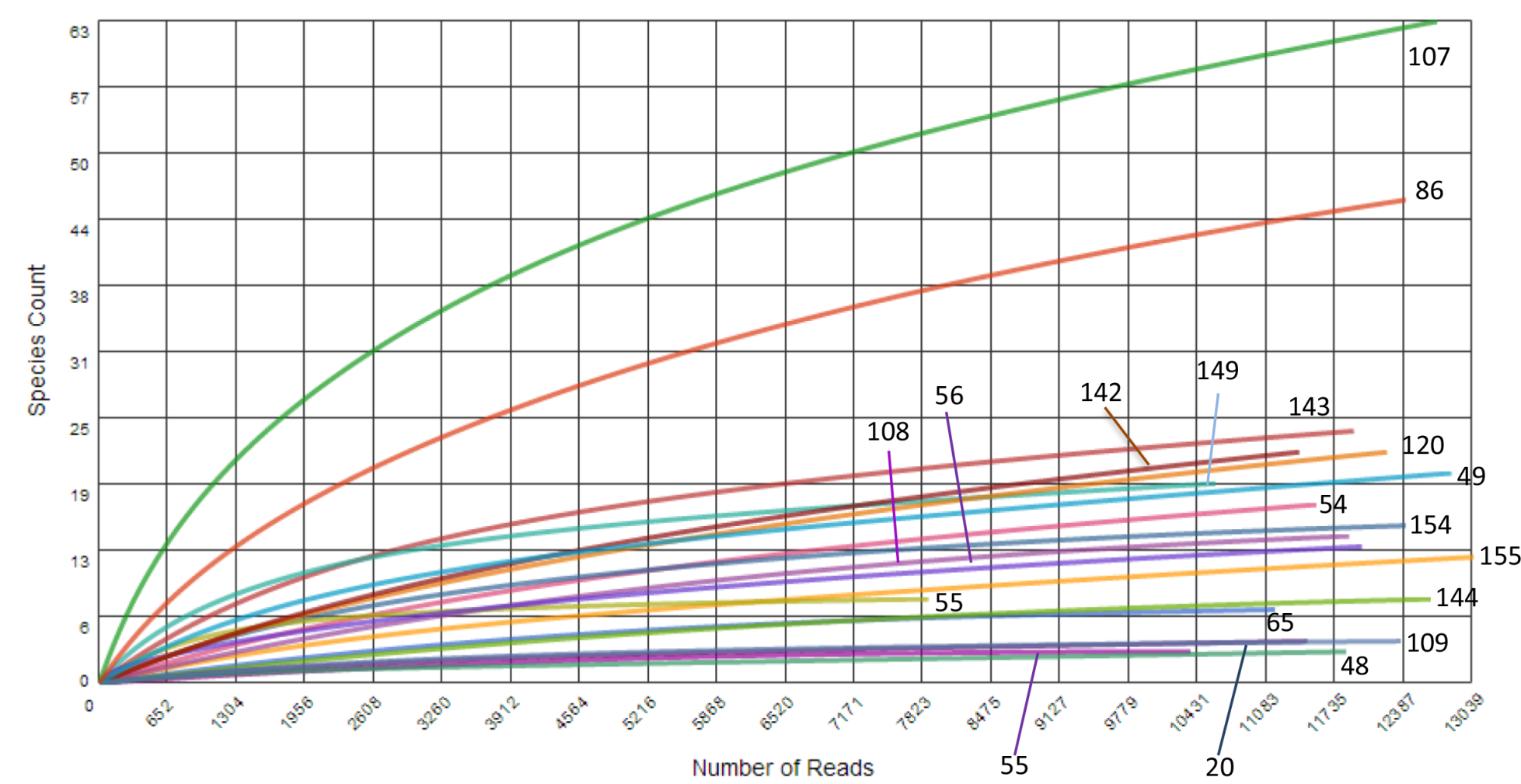

Supplement: S2 Fig — (TIF) [file pone.0135756.s002.tif]

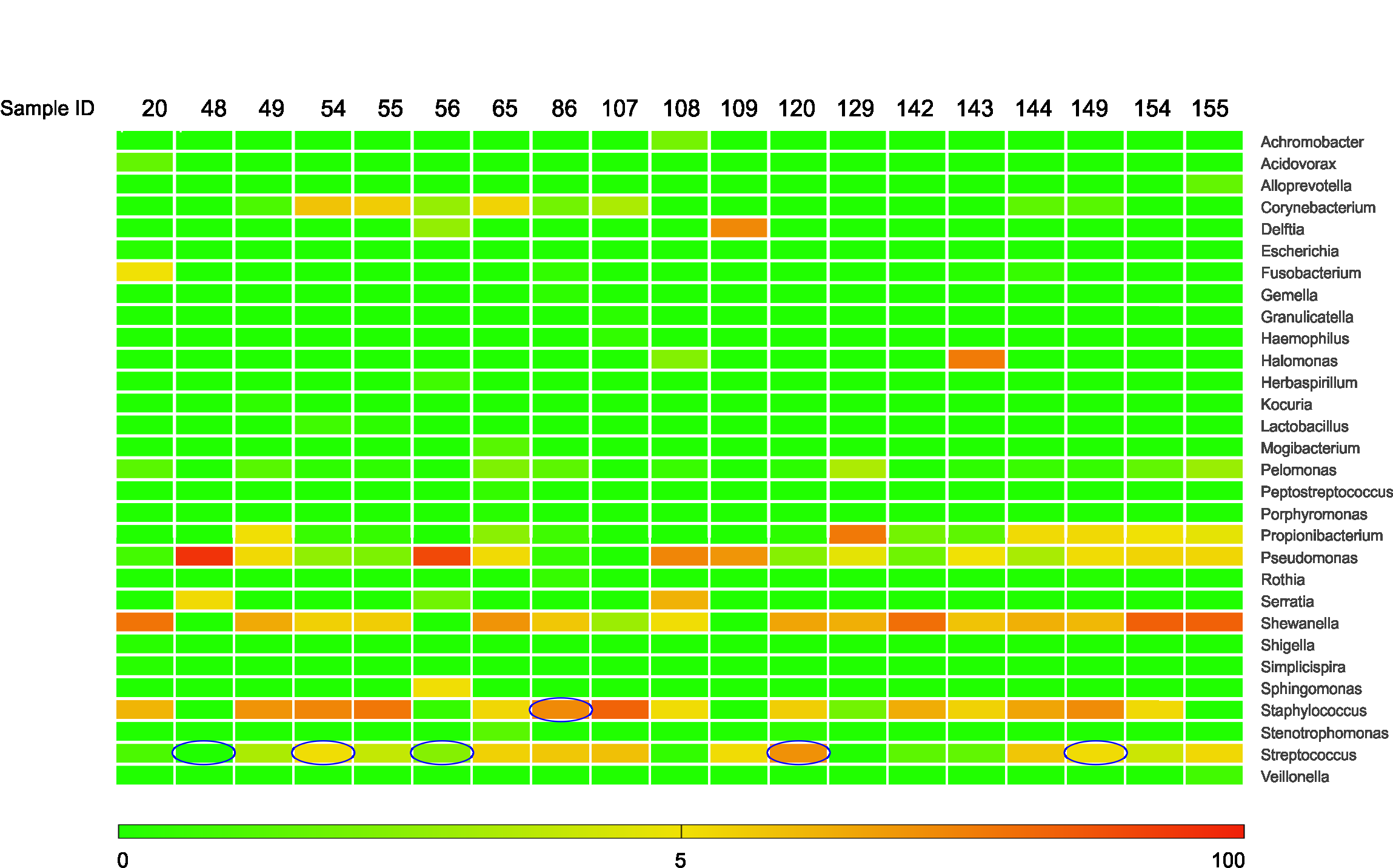

Supplement: S3 Fig — Blue ellipses indicate culture results. (TIF) [file pone.0135756.s003.tif]
