# Supplementary material for: Bacterial Landscape of Bloodstream Infections in Neutropenic Patients via High Throughput Sequencing
Source: PLoS One. 2015 Aug 13;10(8):e0135756. doi: 10.1371/journal.pone.0135756 (PMC4536222; doi:10.1371/journal.pone.0135756)
Supplement: S2 Table — 1 In patients 9 and 25, sequencing identified S. mitis, S. pneumoniae and S. pseudopneumoniae, while, apart from these three species, in patient 27 S. infantis, S. oralis and S. australis were also detected, confirming the presence of viridans streptococci. 2In patient 10, S. dysgalactiae (β-hemolytic streptococci) was detected by sequencing. 3In patient 17, in agreement with the culture result, sequencing detected a coagulase-negative Staphylococcus (S. Saphrophyticus). CoNS: Coagulase-negative staphylococci. (DOCX) [file pone.0135756.s005.docx]

|  | **Sample type** | **Sequencing** | **Culture** |
| --- | --- | --- | --- |
| Patient 2 | Persisting fever | Shewanella | - |
|  |  | Staphylococcus |  |
|  |  | Fusobacterium |  |
|  |  | Acidovorax |  |
|  |  | Pelomonas |  |
| Patient 7 | Fever onset | Pseudomonas | Viridans streptococci |
|  |  | Serratia |  |
|  |  | Delftia |  |
| Patient 7 | Follow up | Staphylococcus | - |
|  |  | Shewanella |  |
|  |  | Pseudomonas |  |
|  |  | Propionibacterium |  |
|  |  | Streptococcus |  |
| Patient 9 | Fever onset | Staphylococcus | Viridans streptococci |
|  |  | Corynebacterium |  |
|  |  | Shewanella |  |
|  |  | Streptococcus^1^ |  |
|  |  | Pseudomonas |  |
| Patient 9 | Persisting fever | Staphylococcus | - |
|  |  | Corynebacterium |  |
|  |  | Shewanella |  |
|  |  | Streptococcus |  |
|  |  | Pseudomonas |  |
| Patient 10 | Fever onset | Pseudomonas | Viridans streptococci |
|  |  | Sphingomonas |  |
|  |  | Corynebacterium |  |
|  |  | Delftia |  |
|  |  | Streptococcus^2^ |  |
| Patient 12 | Follow up | Shewanella | NA |
|  |  | Streptococcus |  |
|  |  | Corynebacterium |  |
|  |  | Staphylococcus |  |
|  |  | Pseudomonas |  |
| Patient 15 | Persisting fever | Staphylococcus | - |
|  |  | Streptococcus |  |
|  |  | Corynebacterium |  |
|  |  | Shewanella |  |
|  |  | Haemophilus |  |
| Patient 17 | Persisting fever | Staphylococcus^3^ | CoNS |
|  |  | Streptococcus | Enterococcus faecalis |
|  |  | Shewanella |  |
|  |  | Corynebacterium |  |
|  |  | Pelomonas |  |
| Patient 18 | Fever onset | - | Escherichia coli |
| Patient 21 | Fever onset | Delftia | - |
|  |  | Pseudomonas |  |
|  |  | Streptococcus |  |
|  |  | Propionibacterium |  |
| Patient 23 | Fever onset | Pseudomonas | - |
|  |  | Serratia |  |
|  |  | Staphylococcus |  |
|  |  | Shewanella |  |
|  |  | Halomonas |  |
| Patient 25 | Fever onset | Streptococcus^1^ | Viridans streptococci |
|  |  | Shewanella |  |
|  |  | Staphylococcus |  |
|  |  | Pseudomonas |  |
|  |  | Propionibacterium |  |
| Patient 25 | Persisting fever | - | Viridans streptococci |
| Patient 25 | Persisting fever | Propionibacterium | - |
|  |  | Shewanella |  |
|  |  | Pseudomonas |  |
|  |  | Pelomonas |  |
|  |  | Staphylococcus |  |
| Patient 26 | Follow up | Halomonas | NA |
|  |  | Shewanella |  |
|  |  | Staphylococcus |  |
|  |  | Pseudomonas |  |
|  |  | Streptococcus |  |
| Patient 27 | Fever onset | Staphylococcus | Viridans streptococci |
|  |  | Shewanella |  |
|  |  | Propionibacterium |  |
|  |  | Streptococcus^1^ |  |
|  |  | Pseudomonas |  |
| Patient 27 | Persisting fever | Shewanella | - |
|  |  | Pseudomonas |  |
|  |  | Staphylococcus |  |
|  |  | Propionibacterium |  |
|  |  | Streptococcus |  |
| Patient 29 | Fever onset | - | Viridans streptococci |
| Patient 30 | Fever onset | Shewanella | - |
|  |  | Staphylococcus |  |
|  |  | Propionibacterium |  |
|  |  | Pseudomonas |  |
|  |  | Streptococcus |  |
| Patient 31 | Fever onset | Staphylococcus | - |
|  |  | Shewanella |  |
|  |  | Streptococcus |  |
|  |  | Propionibacterium |  |
|  |  | Pseudomonas |  |
| Patient 32 | Persisting fever | Shewanella | - |
|  |  | Pseudomonas |  |
|  |  | Streptococcus |  |
|  |  | Propionibacterium |  |
|  |  | Pelomonas |  |
| Patient 33 | Fever onset | - | Staphylococcus epidermis |
