# Supplementary material for: Bacterial Landscape of Bloodstream Infections in Neutropenic Patients via High Throughput Sequencing
Source: PLoS One. 2015 Aug 13;10(8):e0135756. doi: 10.1371/journal.pone.0135756 (PMC4536222; doi:10.1371/journal.pone.0135756)
Supplement: S3 Table — (DOCX) [file pone.0135756.s006.docx]

| Sample ID | Total nr of reads | Nr reads after filtering and merging | Chimeric | OTU assigned |
| --- | --- | --- | --- | --- |
| 20 | 466,652 | 273,794 | 20,444 | 132,667 |
| 48 | 474,277 | 273,969 | 36,726 | 121,854 |
| 49 | 795,628 | 433,277 | 26,279 | 166,931 |
| 54 | 523,375 | 257,355 | 26,182 | 128,237 |
| 55 | 364,535 | 149,576 | 21,246 | 76,419 |
| 56 | 475,885 | 286,183 | 30,297 | 85,901 |
| 65 | 383,000 | 186,300 | 13,523 | 74,459 |
| 86 | 584,211 | 315,811 | 46,292 | 132,412 |
| 107 | 629,698 | 319,846 | 42,250 | 143,208 |
| 108 | 717,971 | 396,013 | 53,756 | 227,974 |
| 109 | 594,559 | 319,827 | 26,067 | 141,713 |
| 120 | 799,542 | 439,307 | 57,150 | 213,283 |
| 129 | 574,904 | 288,058 | 7,697 | 92,281 |
| 142 | 681,706 | 379,007 | 37,799 | 214,555 |
| 143 | 659,540 | 344,781 | 36,145 | 172,019 |
| 144 | 632,071 | 333,763 | 28,610 | 136,684 |
| 149 | 607,915 | 314,855 | 40,279 | 173,335 |
| 154 | 687,741 | 366,034 | 44,607 | 184,111 |
| 155 | 737,094 | 407,992 | 33,912 | 146,549 |
| Total | 11,390,304 | 6,085,748 | 629,261 | 2,764,592 |
| Average per sample | 599,489 | 320,302 | 33,119 | 145,504 |
