# Supplementary material for: Bacterial Landscape of Bloodstream Infections in Neutropenic Patients via High Throughput Sequencing
Source: PLoS One. 2015 Aug 13;10(8):e0135756. doi: 10.1371/journal.pone.0135756 (PMC4536222; doi:10.1371/journal.pone.0135756)
Supplement: Supplementary file 4 [file pone.0135756.s004.docx]

	Fever	Persisting fever	Follow up 1	Follow up 2	Follow up 3
Patient 1	- / -		- /na	- / -	NA
Patient 2		+ / -	NA	NA	NA
Patient 3-1	- / -		- /na	NA	NA
Patient 3-2	- / -		- /na	- /na	NA
Patient 4-1	- / -		- /na	- /na	NA
Patient 4-2		- / -	NA	NA	NA
Patient 4-3	- / -		- /na	- /na	NA
Patient 5-1	- / -		- /na	NA	NA
Patient 5-2		- / -	NA	NA	NA
Patient 6		- / -	NA	NA	NA
Patient 7	+ / +		+ / na	- / -	NA
Patient 8		- / -	NA	NA	NA
Patient 9	+ / +	+ / -	- /na	- /na	NA
Patient 10	+ / +		NA	NA	NA
Patient 11-1		- / -	NA	NA	NA
Patient 11-2		- / -	NA	NA	NA
Patient 11-3	- / -		NA	NA	NA
Patient 12-1	- / -	- / -	+ /na	- /na	NA
Patient 12-2	- / -		NA	NA	NA
Patient 13-1	- / -	- / -	- /na	NA	NA
Patient 13-2		- / -	NA	NA	NA
Patient 13-3	- / -		- /na	- /na	- /na
Patient 14	- / -		NA	NA	NA
Patient 15-1	- / -		- /na	- /na	- /na
Patient 15-2	- / -	- / -	- /na	- /na	NA
Patient 15-3	- / -	+ / -	NA	NA	NA
Patient 16		- / -	NA	NA	NA
Patient 17		+ / +	NA	NA	NA
Patient 18	- / +		NA	NA	NA
Patient 19-1	- / -	- / -	- /na	NA	NA
Patient 19-2	- / -	- / -	- /na	NA	NA
Patient 20	- / -		NA	NA	NA
Patient 21-1	+ / -		NA	NA	NA
Patient 21-2	- / -		- /na	NA	NA
Patient 22-1	- / -		NA	NA	NA
Patient 22-2	- / -		NA	NA	NA
Patient 23	+ / -		NA	NA	NA
Patient 24	- / -		- /na	NA	NA
Patient 25-1	+ / +	- / +	- /na	NA	NA
Patient 25-2		+ / -	NA	NA	NA
Patient 26-1	- / -		- /na	- /na	- / -
Patient 26-2	- / -		- /na	- /na	+ / na
Patient 27-1		- / -	NA	NA	NA
Patient 27-2	+ / +	+ / -	- /na	NA	NA
Patient 28-1	- / -		NA	NA	NA
Patient 28-2		- / -	NA	NA	NA
Patient 28-3		- / -	NA	NA	NA
Patient 29	- / +		- /na	- /na	NA
Patient 30	+ / -		NA	NA	NA
Patient 31	+ / -		NA	NA	NA
Patient 32	- / -	+ / -	- /na	NA	NA
Patient 33	- / +		- /na	NA	NA
